# Bone marrow mesenchymal stem cell-derived vascular endothelial growth factor attenuates cardiac apoptosis via regulation of cardiac miRNA-23a and miRNA-92a in a rat model of myocardial infarction

**DOI:** 10.1371/journal.pone.0179972

**Published:** 2017-06-29

**Authors:** Yi-Sun Song, Hyun-Woo Joo, In-Hwa Park, Guang-Yin Shen, Yonggu Lee, Jeong Hun Shin, Hyuck Kim, Kyung-Soo Kim

**Affiliations:** 1 Graduate School of Biomedical Science and Engineering, Hanyang University, Seoul, South Korea; 2 Department of Internal Medicine, Hanyang University College of Medicine, Seoul, South Korea; 3 Department of Internal Medicine, Hanyang University Guri Hospital, Guri, South Korea; 4 Department of Thoracic and Cardiovascular Surgery, Hanyang University College of Medicine, Seoul, South Korea; University of Cincinnati College of Medicine, UNITED STATES

## Abstract

Bone marrow-mesenchymal stem cell (BM-MSC) therapy improves the recovery of cardiac function after myocardial infarction (MI); however, the underlying molecular mechanisms are not completely understood. Recent studies have shown that microRNAs (miRNAs) modulate the pathophysiology of cardiovascular diseases. Here, we investigated the mechanisms underlying the effects of BM-MSC-derived paracrine factors and cardiac miRNAs on myocardial regeneration after MI. In our study, MI was induced by permanent ligation of the left anterior descending (LAD) coronary artery. BM-MSCs transplanted in infarcted rats significantly downregulated the expression of miRNA-23a and miRNA-92a and inhibited apoptosis in the myocardium. An in vitro experiment showed that supernatant from BM-MSCs cultured under hypoxia contained higher levels of vascular endothelial growth factor (VEGF) than that from BM-MSCs under normoxia. In addition, inhibition of miRNA-23a and miRNA-92a reduced cardiac apoptosis. Moreover, the VEGF-containing BM-MSC supernatant inhibited miRNA-23a and miRNA-92a expression and reduced apoptotic signaling in cardiomyocytes under hypoxia. These effects were inhibited when the supernatant was treated with neutralizing antibodies against VEGF. Our results indicate that the paracrine factor, VEGF, derived from transplanted BM-MSCs, regulated the expression of miRNAs such as miRNA-23a and miRNA-92a and exerted anti-apoptotic effects in cardiomyocytes after MI.

## Introduction

Although the mortality rate of myocardial infarction (MI) has much improved since rapid revascularization of occluded coronary arteries became common practice, MI remains to be one of the leading causes of death and chronic heart failure [1]. Stem cell therapy has been recognized as a promising treatment option to restore damaged myocardium after MI [2, 3]. So far, various types of stem cells including mesenchymal stem cells (MSCs) [4, 5], cardiac stem cells [6], bone marrow (BM) stem cells [7], and amniotic stem cells [8] have been reported to reduce infarct size and improve myocardial function after MI; however, mechanisms underlying the effects of stem cell therapies remain unclear.

Paracrine actions of stem cell-derived factors have been recognized as a more important mechanism than direct regeneration of myocardium by the implanted stem cells [9]. MSC therapy has been reported to reduce infarct size through the anti-apoptotic effects of paracrine factors derived from BM-MSCs [10, 11]. We have also reported that amniotic stem cell therapy reduced infarct size and improved cardiac function by reducing apoptosis in infarct myocardium through paracrine actions of stem cell-derived factors [12].

MicroRNAs (miRNAs) are small non-coding RNAs that bind to complementary sequences on mRNAs and regulate many biological processes. Many miRNAs are known to be involved in the pathophysiology of various cardiac diseases and the repair and regeneration of cardiac tissues [13]. In recent studies, miRNAs, including miRNA-15b [14], miRNA-34a [15], miRNA-92a [16], and miRNA-320 [17] have been reported to be involved in the regulation of cardiomyocyte apoptosis after MI. Given that paracrine factors exert anti-apoptotic effects, there may be a link between the actions of paracrine factors from transplanted stem cells and the roles of miRNAs in stem cell therapies for MI. In addition, even though a few potential mechanisms have been proposed for beneficial effects of stem cell therapies [18], there have been no reports related to the role of miRNAs in paracrine effect of transplanted MSC.

Therefore, we hypothesized that reductions in apoptosis and fibrosis of the myocardium in MI after MSC therapy could be associated with the regulation of cardiac miRNA by MSC-released paracrine factors. In this study, we sought to confirm the therapeutic effect of MSCs in a rat model of MI, screened in vitro MSC-released paracrine factors under hypoxic conditions, and described cardiac miRNA regulation by MSC-released paracrine factors.

## Materials and methods

### Animals

All experimental procedures were performed in accordance with the ARRIVE guidelines for research [19], and the Hanyang University Institutional Animal Care and Use Committee approved all protocols (2015-0054A). Male Sprague-Dawley rats (Koatech, Kyungki-do, South Korea), eight weeks old and weighing 200–250 g, were used in this experiment. The animals were maintained in the Hanyang University Medical School Animal Experiment Center and were kept in a specific pathogen-free facility at a controlled temperature (23 ± 2°C) and humidity (55 ± 5%) with a 12 h artificial light-dark cycle.

### Myocardial infarction and cell transplantation

Myocardial infarction (MI) was induced by permanent ligation of the left anterior descending (LAD) coronary artery as previously described [12, 20, 21]. In order to induce MI, rats were anesthetized with a cocktail of tiletamine and zolazepam (Zoletil 100, Virbac, France; dosage 40 mg/kg, i.p.). The chest was opened via lateral thoracotomy, and the heart was exposed using a left anterior thoracotomy. The LAD was ligated with 6–0 polypropylene (Prolene ®; Ethicon, Hamburg, Germany) just below the tip of the left auricle. Ten days after inducing the MI, the rats were randomly divided into two groups (n = 4–5). After anesthesia, one group was intra-myocardial injected with BM-MSCs (PT-2501; Lonza, Walkersville, MD, USA) from passage 4 to 6 while the other group received a saline injection. Sham-operated rats were subjected to similar surgical procedures. Cyclosporin A (5 mg/kg/day i.p., CIPOL®, Chong Kun Dang, Seoul, Korea) was administered from 2 days pre-transplantation until sacrifice [22]. To trace the fate of the transplanted cells, the BM-MSCs were labeled with chloromethyl-benzamidodialkylcarbocyanine (Cell Tracker™: CM-DiI; Molecular Probes, Eugene, OR, USA), according to the manufacturer’s protocol [23]. A total of 1 million BM-MSCs were resuspended in 100 μl phosphate-buffered saline (PBS; WELGENE, Gyeongsangbuk-do, South Korea) and transplanted intramuscularly at three different sites 2 mm apart from the border towards the center of the infarct and from the lateral left ventricle to the septum. Penicillin (500,000 U/ml i.p., Penicillin G potassium, Kun Wha Pharmaceutical Co., Seoul, Korea) was administered after each procedure. Rats were sacrificed under anesthesia with a cocktail of tiletamine and zolazepam (40 mg/kg, i.p.) at 3 days or 4 weeks after treatment with BM-MSCs or saline. Heart tissues including the infarct zone and border zone were collected and either snap-frozen in liquid nitrogen or fixed in 10% formalin solution (pH 7.4).

### Masson’s trichrome staining and TUNEL assay

For histopathology, fixed heart tissues were embedded in paraffin, and cut into 4 μm sections. Collagen in myocardium sections was stained using the Masson's trichrome method. The infarct area was measured as the percent ratio (%) of the injured area, divided by the whole myocardium area in each rat [8]. Apoptotic cells in myocardium sections were stained using a terminal deoxynucleotidyl transferase dUTP nick end labeling (TUNEL) assay with an In Situ Cell Death Detection kit (Roche, Mannheim, Germany). The stained sections were photographed using a light microscope (Leica DM 4000B). Apoptotic nuclei were counted in order to calculate the apoptotic index (number of labeled nuclei / number of total nuclei) in randomly selected five fields from the border zone of each slide [24].

### Isolation and culture of neonatal rat cardiomyocytes

Primary cultures of the cardiomyocytes were established as described previously [25]. In brief, two-days old neonatal rats were anesthetized by inhalation with isoflurane. Heart tissues were quickly cut, and then the ventricles were obtained and washed with PBS. Subsequently, the ventricles were cut into small fragments and digested repeatedly in Hanks’ balanced salt solution (HBSS; Sigma-Aldrich, St. Louis, MO, USA) (without Ca^2+^, Mg^2+^) supplemented with 0.5 mg/ml collagenase type 2 (Worthington, Lakewood, NJ, USA) and 20 mM HEPES (Sigma-Aldrich Chemie GmbH, Steinheim, Germany). After being centrifuged at 1000 rpm for 5 min, the collected cells were passed through a cell strainer (40 mm, BD Falcon, Bedford, MA, USA) and seeded onto uncoated 100-mm plastic dishes for 1 h 30 min at 37°C in a humidified atmosphere with 5% CO_2_. The non-adherent cardiomyocytes were seeded on six well coated plates. After 24 h, the cardiomyocytes were washed with PBS and cultured in Dulbecco's modified eagle's medium (DMEM; Gibco, Grand Island, NY, USA) with 20% fetal bovine serum (FBS; Gibco), 100 units/ml penicillin (Gibco).

### Preparation of BM-MSC-conditioned media and luminex analysis

To obtain BM-MSC-conditioned media, BM-MSCs at passage 4 to 6 were cultured for 48 h in MSC growth medium (PT-3001; Lonza) with 10% FBS, rinsed with serum-free medium and cultured for 48 h in serum-free DMEM media under either normal or hypoxic conditions. The hypoxic condition was induced using an MIC-101 modular incubator chamber (Billups-Rothenberug, Del Mar, CA, USA), that was flushed with 95% N2 and 5% CO2 and placed at 37°C for up to 48 h, as indicated. The conditioned media were collected, centrifuged and stored frozen at -75°C. The collected medium was used for Luminex analysis and other in vitro experiments. The conditioned media from the BM-MSCs under normal and hypoxic conditions were analyzed using a Magnetic Luminex screening assay (R&D Systems, Abingdon, UK), according to the manufacturer's instructions [26].

### Annexin V staining

Apoptotic cells were quantified using the Fluorescin isothiocyanate (FITC) annexin V apoptosis detection kit 1 (BD Biosciences Pharmingen, San Diego, CA, USA), according to the manufacturer's instructions [27]. The trypsinized cells were stained with FITC annexin V and propidium iodide (PI) and then analyzed by flow cytometry using a BD FACS Canto (BD Biosciences, San Jose, CA, USA). The total area of annexin V positive cardiomyocytes was determined by computerized morphometry (BD FACS Diva software).

### RNA isolation, miRNA microarray, and real-time PCR with TaqMan probes

Total RNA was harvested from heart tissue and cardiomyocytes using Qiazol reagent (Qiagen, Valencia, CA, USA), according to the manufacturer’s instructions. The concentration of each sample was measured using a Nanodrop ND-2000 spectrophotometer (Thermo Fisher Scientific Inc., Wilmington, DE, USA). For miRNA microarray analysis, miRNA expression profiling was conducted by miRNA microarray analysis using the miRCURY LNA™ miRNA Array (Exiqon, Vedbaek, Denmark) containing 700 mature rat miRNAs. The accuracy of the microarray data was validated using real-time PCR with TaqMan probes. For real-time PCR analysis, total RNA was reverse transcribed with stem-loop primers and the TaqMan® MicroRNA Reverse Transcription kit (Applied BioSystems, Foster City, CA, USA), according to the manufacturer’s instructions [28]. Real-time PCR was performed in duplicate using the TaqMan® MicroRNA assay kit and TaqMan Universal PCR MasterMix (Applied Biosystems) for miRNA-23a and miRNA-92a, according to the manufacturer’s instructions. Real-time PCR was performed using the LightCycler® 480 program (Roche) for 40 cycles, (10 s each, at 95°C, 60°C, 72°C). Relative miRNA expression levels were normalized using the RNU6B (U6) small non-coding RNA as an endogenous control. The information of TaqMan® MicroRNA assay kit is shown in S1 Table.

### Transfection of miRNA inhibitor

To identify the function following knock-down of miRNA-23a and miRNA-92a expression, primary cultured cardiomyocytes were cultured in a six-well plate and were incubated with 40 nM miRNA-23a and miRNA-92a inhibitors (GenePharma, Shanghai, China) using INTERFERin transfection agent (PolyPlus-transfection SA, Illkirch, France), according to the manufacturer’s instructions [29]. For control transfections, 40 nM of negative control inhibitor was used. After a 12 h transfection, serum-free medium was added, and the cells were cultured under normoxic or hypoxic conditions for 48 h. The efficiency of the miRNA inhibitor transfection was determined by real-time PCR, performed as described above. The sequences of inhibitors are represented in S2 Table.

### Neutralizing antibodies against paracrine factors

To confirm the effects of the paracrine factors vascular endothelial growth factor (VEGF) and monocyte chemoattractant protein (MCP)-1, the action of these paracrine factors present in BM-MSC-conditioned media was blocked using a neutralizing antibody (VEGF, 50 ng/ml; MCP-1, 500 ng/ml; R&D Systems) [30]. Cardiomyocytes were cultured in serum-free medium in six-well plates to induce starvation for 24 h prior to use. The paracrine factor-neutralizing antibodies were added to the BM-MSC-conditioned media and incubated for a period of 1 h prior to being added to the cardiomyocytes. After 48 h, the cells were washed with cold PBS and prepared for real-time PCR and annexin V staining.

### Statistical analysis

Data are expressed as the mean ± standard deviation (SD). Data were analyzed using Student’s t-test (for single comparisons) or one-way ANOVA (for multiple comparisons), and post-hoc multiple comparisons were made with Tukey’s test using Statistical Program for the Social Sciences (SPSS software version 21.0 (SPSS, Inc., Chicago, IL, USA)). A P-value of 0.05 was considered statistically significant.

## Results

### BM-MSC therapy improves myocardiac infarction

**Fig 1:**
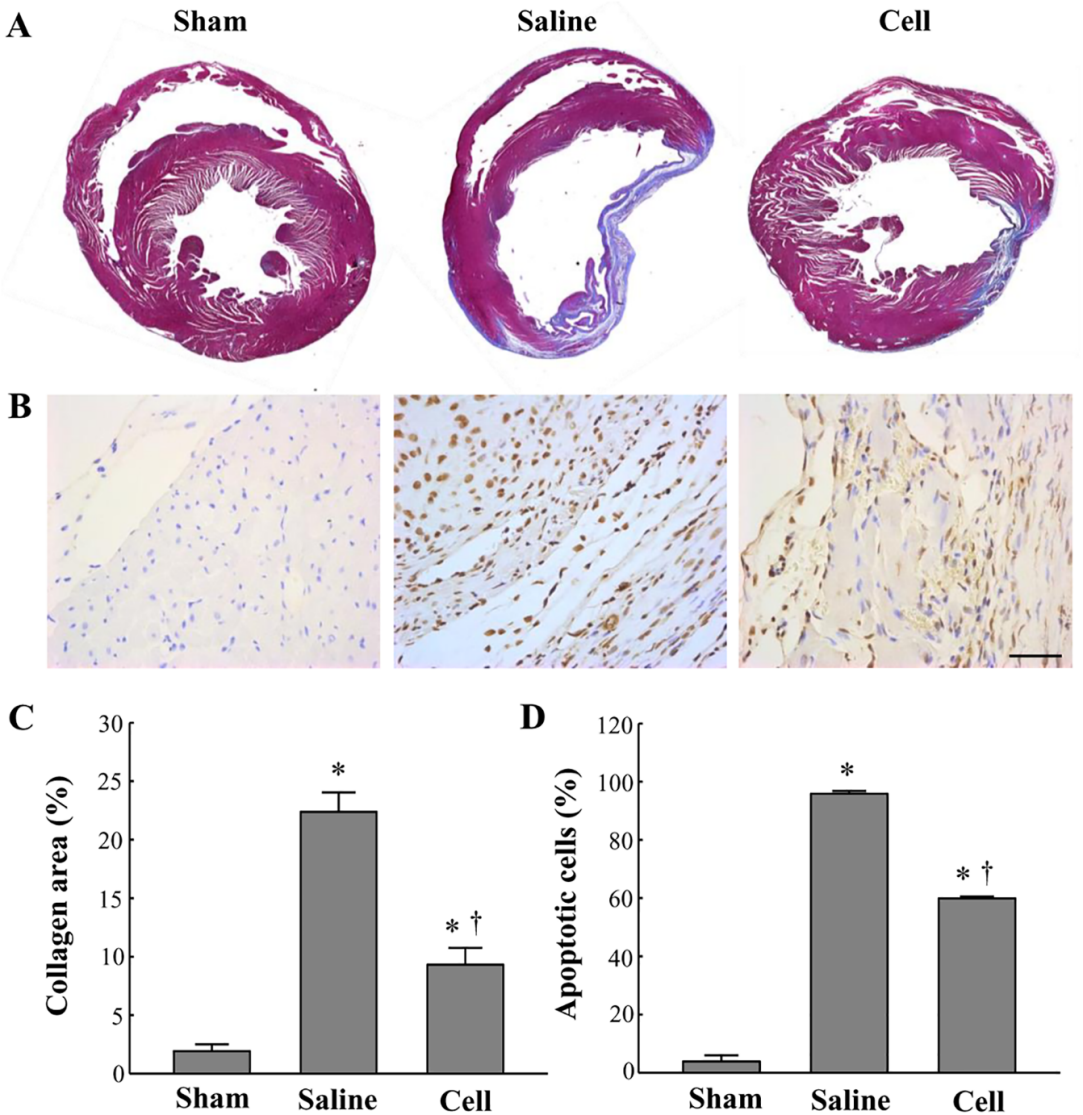
BM-MSC therapy improves fibrosis and apoptosis in a rat model of MI. (A) Representative images of Masson's trichrome staining of whole heart tissue at 4 weeks after treatment for each group. (B) Representative photomicrographs showing TUNEL assay in the peri-infarct region at 4 weeks after treatment for each group. Scale bar = 50 μm. (C) Results of quantitative analysis of collagen area as ratio of fibrotic area to whole heart area. (D) Results of quantitative analysis of apoptotic cells. Sham, surgical procedure with no induction of MI; Saline, saline treatment after induction of MI; Cell, cell treatment after induction of MI. All data are expressed as mean ± SD (n = 5 per group). *P < 0.05 vs. sham control group. †P < 0.05 vs. saline group.

In 4 weeks following cell transplantation, immunofluorescence analysis confirmed the presence of CM-DiI-positive BM-MSCs within the infarcted region of the rat myocardium (S1 Fig). Infarct sizes were smaller in the BM-MSC-treated group than in the saline-treated group (Fig 1A). The level of apoptosis and fibrosis in the myocardium was significantly lower in the BM-MSC-treated group than in the saline-treated group (Fig 1B–1D and S2 Fig).

### BM-MSCs transplantation regulates cardiac miRNAs

**Fig 2:**
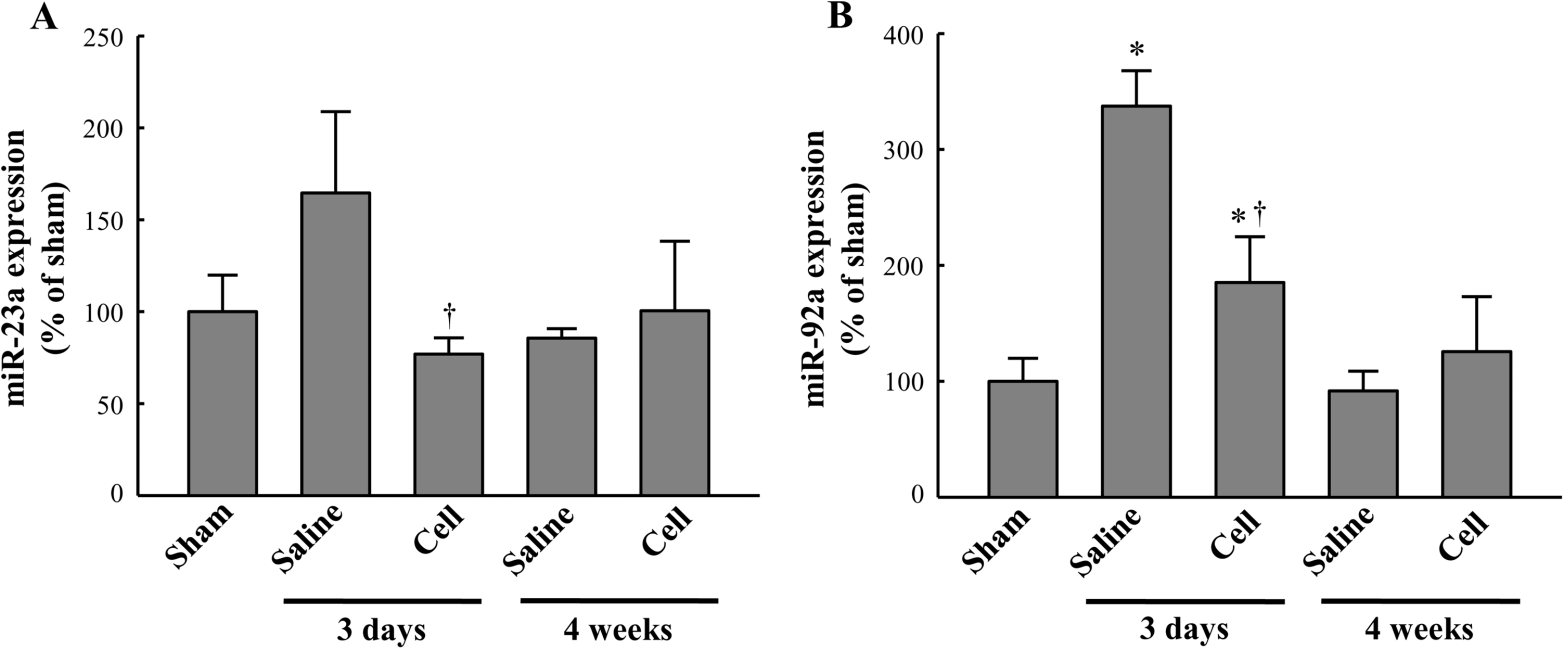
BM-MSC therapy regulates cardiac miRNAs in a rat model of MI. MiRNA expression was measured in the peri-infarct region by real-time PCR using TaqMan probes at 3 days or 4 weeks after treatment. MiRNA-23a (A) and miRNA-92a (B) expression in response to treatment with BM-MSCs in comparison with saline or sham at 3 days or 4 weeks after treatment. All data are expressed as mean ± SD (n = 5 per group). *P < 0.05 vs. sham control group. †P < 0.05 vs. saline group at 3 days.

To identify miRNAs whose expression levels markedly changed after BM-MSCs transplantation, we transplanted BM-MSCs into an infarcted heart and analyzed the expression of miRNA by miRNA microarray 3 days after treatment with BM-MSCs or saline. The results of the miRNA microarray revealed that five miRNAs were expressed at significantly higher levels in the BM-MSC-treated group than in the saline-treated group, whereas 34 other miRNAs were present at significantly lower levels in the BM-MSC-treated group than in the saline-treated group (S3 Fig). Among these miRNAs, we selected candidate miRNAs known to be associated with cardiac diseases. To confirm the accuracy of the microarray data, we investigated the expression levels of miRNAs using real-time PCR with TaqMan probes (S4 Fig). Because the expression of miRNA-23a and miRNA-92a was validated to be consistent with the microarray data and showed simultaneously that highest regulated by BM-MSC treatment in the real-time PCR, we focused on miRNA-23a and miRNA-92a. Indeed, expression of miRNA-23a and miRNA-92a was significantly lower in the BM-MSC-treated group than in the saline-treated group 3 days after treatment, although no significant difference was observed between the BM-MSC-treated group and saline-treated group 4 weeks after treatment (Fig 2A and 2B).

### BM-MSCs release paracrine factors

**Fig 3:**
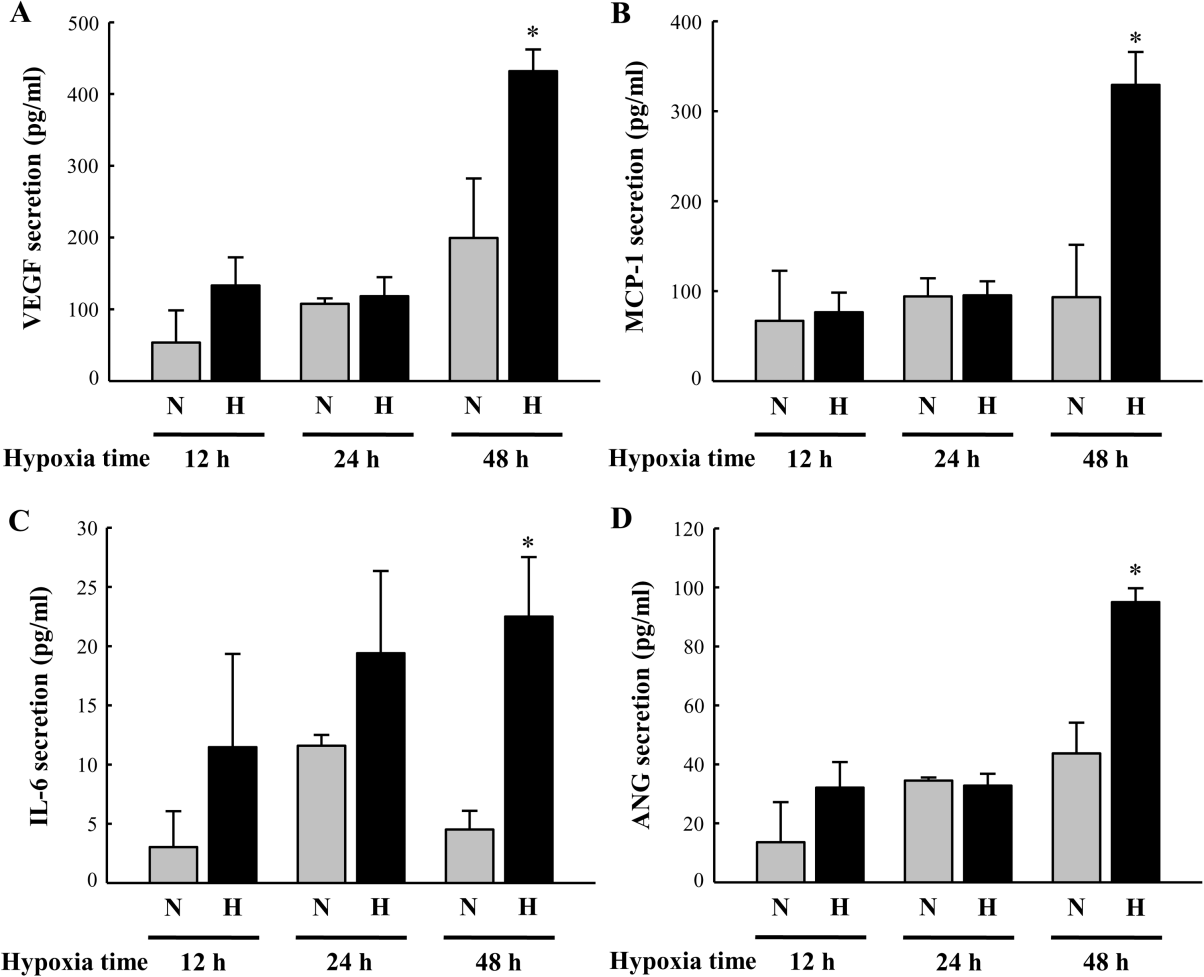
BM-MSC-conditioned media contains paracrine factors when cells are exposed to hypoxia. Paracrine factors were measured in the hypoxia-exposed BM-MSC-conditioned media by Magnetic Luminex assay. VEGF (A), MCP-1 (B), IL-6 (C), and ANG (D) expression in response to hypoxia exposure time in comparison with normoxia. N or H indicate normoxia or hypoxia, respectively. All data are expressed as mean ± SD (n = 5 per group). *P < 0.05 vs. normoxia for 48 h.

To identify paracrine factors released from BM-MSCs, we exposed BM-MSCs to hypoxic conditions and collected the conditioned medium. Using a Magnetic Luminex assay, we investigated the conditioned medium containing paracrine factors such as adiponectin, angiogenin (ANG), brain-derived neurotrophic factor (BDNF), epidermal growth factor (EGF), fibroblast growth factor (FGF)-21, granulocyte-colony stimulating factor (G-CSF), glial cell-derived neurotrophic factor (GDNF), granulocytemacrophage-colony stimulating factor (GM-CSF), hepatocyte growth factor (HGF), interleukin (IL)-6, leptin, MCP-1, and VEGF. Among the above listed paracrine factors, the levels of VEGF, MCP-1, IL-6, and ANG were significantly higher in the 48 h hypoxia-exposed BM-MSC-conditioned media than in media from cells exposed to normoxia (Fig 3A–3D and S3 Table).

### BM-MSC-conditioned media inhibits miRNA-23a and miRNA-92a expression and apoptosis in vitro

**Fig 4:**
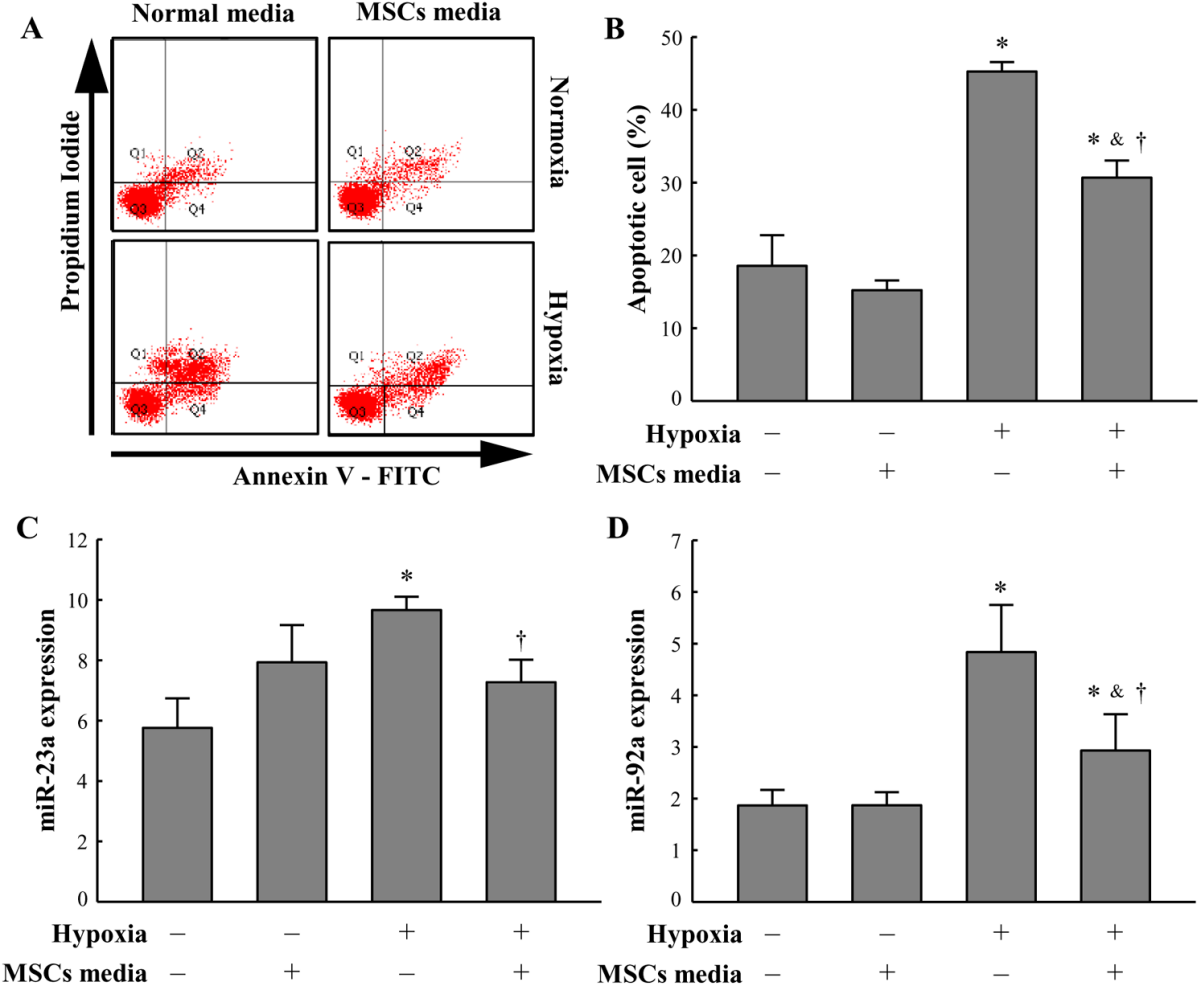
Hypoxia-exposed BM-MSC-conditioned media reduces apoptosis upon exposure of cardiomyocytes to hypoxia for 48 h. (A) Dot plots display the stages of apoptotic death of cardiomyocytes: Annexin−/PI− (Q3), viable cells; Annexin+/PI− (Q4), cells undergoing apoptosis; Annexin+/PI+ (Q2), cells that are in end-stage apoptosis or are already dead; Annexin−/PI+ (Q1), cells that are in necrosis. MSCs media indicates hypoxia-exposed BM-MSC-conditioned media. (B) Quantitative analysis of apoptotic cells (Q2+Q4). Hypoxia-exposed BM-MSC-conditioned media reduces hypoxia-induced miRNA expression in vitro. MiRNA expression was measured by real-time PCR using TaqMan probes. MiRNA-23a (C) and miRNA-92a (D) expression in response to treatment with hypoxia-exposed BM-MSC-conditioned media in comparison without hypoxia-exposed BM-MSC-conditioned media in hypoxia. All data are expressed as mean ± SD (n = 5 per group). *P < 0.05 vs. normoxia without hypoxia-exposed BM-MSC-conditioned media. ^&^P < 0.05 vs. normoxia with hypoxia-exposed BM-MSC-conditioned media. †P < 0.05 vs. hypoxia without hypoxia-exposed BM-MSC-conditioned media.

To confirm whether BM-MSC may inhibit miRNA-23a and miRNA-92a expression in a paracrine manner, we analyzed apoptosis and miRNA expression in vitro. Based on annexin V staining, the number of apoptotic cardiomyocytes was significantly higher in the 48 h hypoxia-exposed cardiomyocytes than in cardiomyocytes exposed to normoxia. Additionally, the number of apoptotic cells was also significantly lower in the hypoxia-exposed cardiomyocytes treated with hypoxia-exposed BM-MSC-conditioned media than in cardiomyocytes not treated with hypoxia-exposed BM-MSC-conditioned media (Fig 4A and 4B). In a real-time PCR assay using TaqMan probes, the expression levels of miRNA-23a and miRNA-92a were significantly higher in the 48 h hypoxia-exposed cardiomyocytes than in the normoxia-exposed cardiomyocytes and were also significantly lower in the hypoxia-exposed cardiomyocytes treated with hypoxia-exposed BM-MSC-conditioned media than in cardiomyocytes not treated with hypoxia-exposed BM-MSC-conditioned media (Fig 4C and 4D). The hypoxia-exposed BM-MSC-conditioned media significantly reduced apoptosis in cardiomyocytes and suppressed the hypoxia-induced up-regulation of miRNA-23a and miRNA-92.

### Cardiac miRNA-23a and miRNA-92a induce cardiac apoptosis

**Fig 5:**
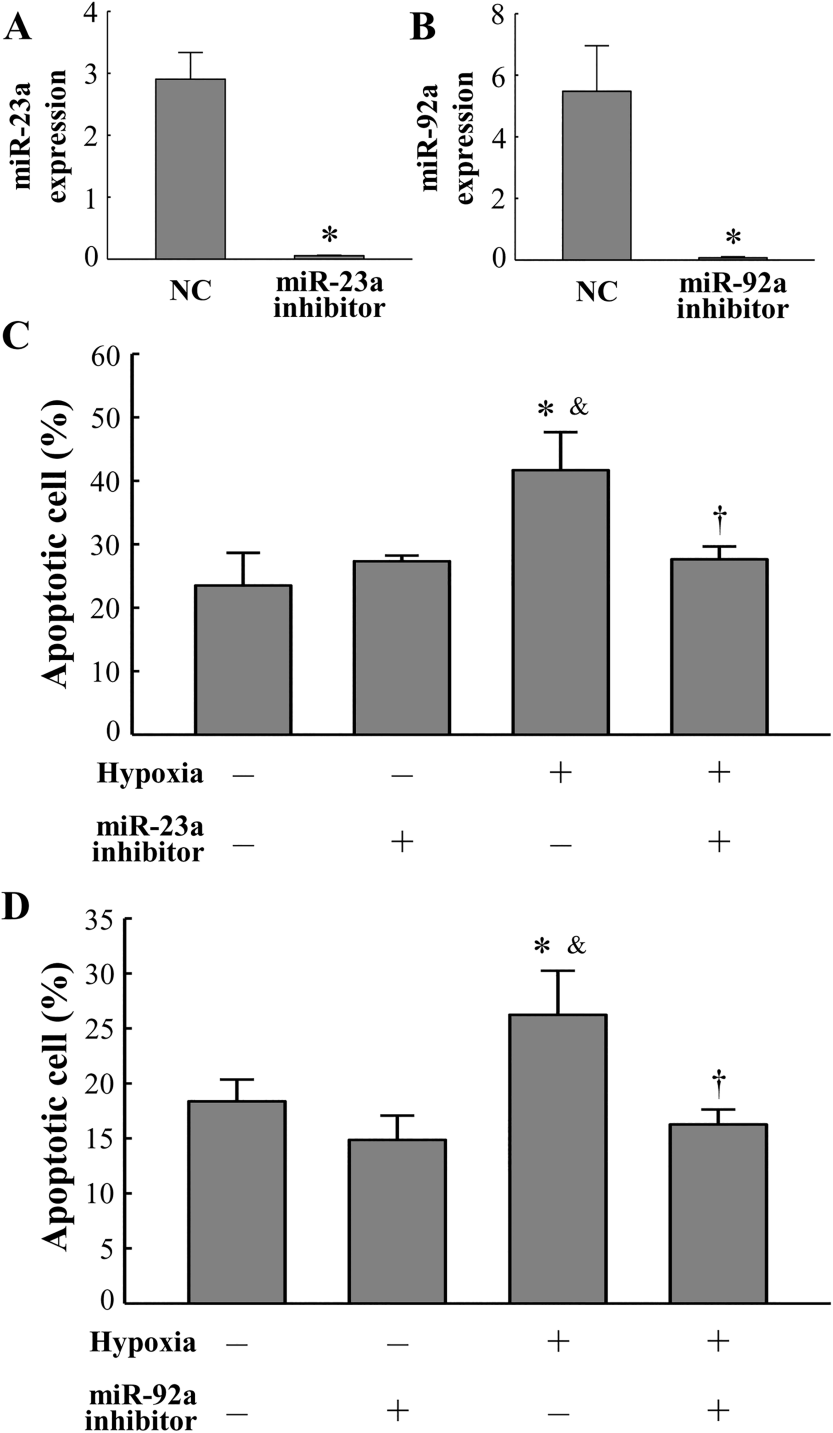
Inhibition of miRNA-23a and miRNA-92a prevents apoptosis of cardiomyocytes. (A, B) Transfection efficiency of the miRNA-23a inhibitor (A) and miRNA-92a inhibitor (B) was determined by real-time PCR using TaqMan probes. Quantitative analysis of apoptotic cells in cardiomyocytes transfected either with miRNA-23a inhibitor (C) or miRNA-92a inhibiton (D). Apoptotic cells were measured by annexin V staining. All data are expressed as mean ± SD (n = 5 per group). *P < 0.05 vs. normoxia without miRNA inhibitor. ^&^P < 0.05 vs. normoxia with miRNA inhibitor. †P < 0.05 vs. hypoxia without miRNA inhibitor.

In cultured cardiomyocytes, the transfection of miRNA-23a and miRNA-92a inhibitors significantly abolished the expression level of miRNA-23a and miRNA-92a (Fig 5A and 5B). To confirm whether cardiac miRNA-23a and miRNA-92a induce cardiac apoptosis, we inhibited miRNA-23a and miRNA-92a by miRNA inhibitor transfection. In the hypoxia-exposed cardiomyocytes, the number of apoptotic cells was significantly lower in the miRNA inhibitor treated group than in the miRNA negative control treated group. Inhibition of the hypoxia-induced upregulation of miRNA-23a and miRNA-92a reduced cardiac apoptosis (Fig 5C and 5D).

### Paracrine factor neutralization inhibits the anti-apoptotic effect of BM-MSCs

**Fig 6:**
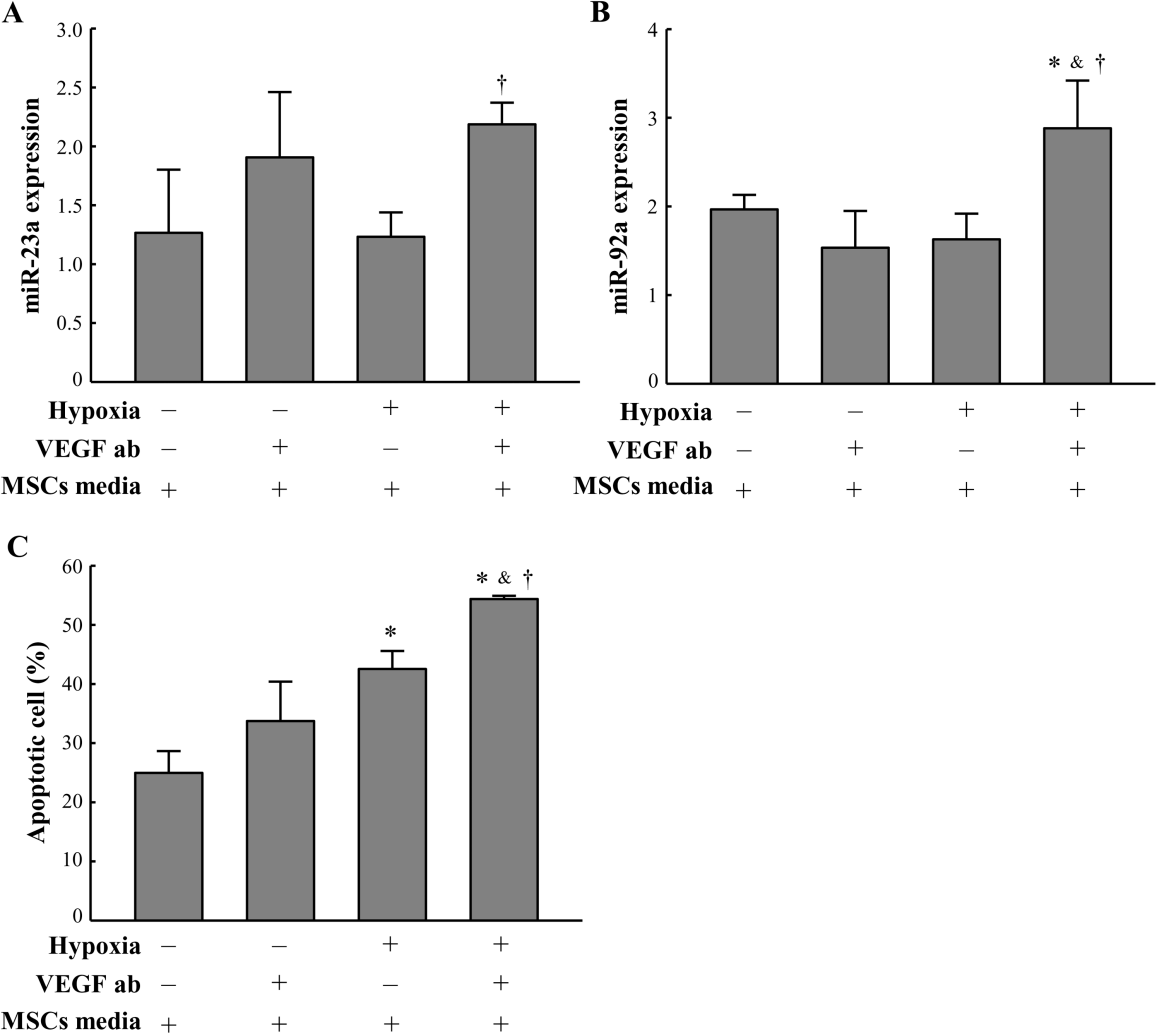
VEGF secreted from BM-MSCs regulates hypoxia-induced miRNA-23a and miRNA-92a expression in vitro. The effects of VEGF secreted from BM-MSCs on hypoxia-induced miRNA-23a (A) and miRNA-92a (B) expression. Expression of the miRNA was determined by real-time PCR using TaqMan probes. (C) Quantitative analysis of apoptotic cells in cardiomyocytes-administered neutralizing antibodies against VEGF (VEGF ab). MSCs media indicates hypoxia-exposed BM-MSC-conditioned media. Apoptotic cells were measured by annexin V staining. All data are expressed as mean ± SD (n = 5 per group). *P < 0.05 vs. normoxia without VEGF ab. ^&^P < 0.05 vs. normoxia with VEGF ab. †P < 0.05 vs. hypoxia without VEGF ab.

To confirm whether paracrine factors derived from BM-MSCs inhibit the hypoxia-induced upregulation of miRNA-23a and miRNA-92a, resulting in reduced cardiac apoptosis, paracrine factors were blocked in hypoxia-exposed BM-MSC-conditioned media using neutralizing antibodies. Of the released paracrine factors that showed significant differences in the hypoxia-exposed BM-MSC-conditioned media compared to the normoxia-exposed conditioned media, VEGF and MCP-1 were selected as candidate factors, because their absolute concentrations were remarkedly higher than those of other paracrine factors (>3-fold higher in VEGF and MCP-1 than in others in the 48 h hypoxia-exposed BM-MSC-conditioned media). The expression levels of miRNA-23a and miRNA-92a were significantly higher in hypoxia-exposed cardiomyocytes treated with VEGF-neutralizing antibody than in those not treated with VEGF-neutralizing antibody. The neutralization of VEGF in hypoxia-exposed BM-MSC-conditioned media showed a significantly reversed influence on the reduced expression of miRNA-23a and miRNA-92a by hypoxia-exposed BM-MSC-conditioned media (Fig 6A and 6B). In addition, the number of apoptotic cardiomyocytes was significantly higher in the hypoxia-exposed cardiomyocytes treated with VEGF-neutralizing antibody than in cells not treated with VEGF-neutralizing antibody. The neutralization of VEGF in hypoxia-exposed BM-MSC-conditioned media significantly reversed the anti-apoptotic effects of hypoxia-exposed BM-MSC-conditioned media on cardiomyocytes (Fig 6C), whereas neutralizing MCP-1 had no effect on miRNA-23a and miRNA-92a expression and apoptosis of cardiomyocytes (S5 Fig).

## Discussion

The results show that VEGF released from transplanted BM-MSCs inhibited the expression of miRNA-23a and miRNA-92a in cardiomyocytes, which led to decrease in apoptosis and fibrosis in the infarcted myocardium. We reproduced the anti-apoptotic effects of BM-MSC therapy on infarcted myocardium. Through a screening microarray experiment, we selected promising candidate miRNAs, miRNA-23a and miRNA-92a, which could explain the mechanisms of the effects of BM-MSC therapy. Further, we created a hypoxic condition to simulate the environment for BM-MSCs in the infarcted myocardium and tested the same miRNAs to be expressed in cardiomyocytes by the paracrine factors released from BM-MSCs.

BM-MSC transplantation is one of the most promising cell therapies in the field of cardiovascular diseases [31]. Its therapeutic effects on MI, including the improvement of cardiac function and the reduction in apoptosis, fibrosis, and eventually infarct sizes, have been well demonstrated in previous animal and human studies [4, 18, 32–35]. Additionally, BM-MSC therapy has been reported to promote cardiomyocyte differentiation [36]. Our results also showed that BM-MSC transplantation reduced infarct sizes, cardiomyocyte apoptosis, and fibrosis in infarcted myocardium.

Mechanisms of action of the therapeutic effects of BM-MSCs for MI remain unclear. However, some studies suggest that paracrine factors derived from BM-MSCs may play important roles in reducing apoptosis in infarcted myocardium [11, 37]. Takahashi et al. reported that paracrine factors from stem cells decide the fate of injured myocardium [7]. Here, we observed MSC-released paracrine factors; among these, VEGF was recognized to regulate the expression levels of pro-apoptotic miRNAs in cardiomyocytes. VEGF has been known to promote angiogenesis of tissues in ischemic conditions [38]; however, VEGF appears to play a role as a paracrine factor in stem cell-mediated myocardial protection [39]. In this regard, VEGF released from transplanted MSCs has also been reported to stimulate endogenous repair mechanisms of cardiomyocytes in a rat MI model [40]. In addition, a previous study demonstrated the therapeutic effects of MSCs through paracrine effect, showing enhanced VEGF levels in heart tissue and serum after MSC transplantation in a mouse MI model [41]. In our study, we confirmed that paracrine factors such as VEGF were released under hypoxic conditions as a result of the anti-apoptotic effect of BM-MSC on cardiomyocytes. This indicates that VEGF plays a critical role in the paracrine mechanisms mediated by BM-MSCs, although direct in vivo evidence regarding the function of BM-MSC-secreted VEGF is required.

MiRNAs are important molecules that regulate many biological processes, pertubations in which cause various diseases [42]. Growing evidence suggests that the dysregulation of miRNA expression in heart tissue is related to the pathogenesis of cardiovascular disease [43, 44]. In particular, a previous study demonstrated that expression of miRNA-92a was up-regulated 24 h after the induction of MI, and that treatment with antagomir-92a after MI improved left ventricular function and reduced cardiac apoptosis at the border areas of the infarction [45]. Moreover, the regional abolishment of miRNA-92a was shown to improve myocardial ischemia/reperfusion injury in a large animal model, suggesting that HL-1 cardiomyocytes treated with miRNA-92a inhibitor are resistant to hypoxia/reoxygenation-induced cell death [16]. In a recent study, Zhang et al. reported that inhibition of miRNA-92a was able to significantly alleviate apoptosis caused by hypoxia/reoxygenation in H9c2 cardiomyocytes [46]. They also identified Smad7 as the target of miRNA-92a; treatment with miRNA-92a inhibitor increased the protein levels of Smad7 although the Smad7 mRNA levels remained unchanged. Additionally, Chhabra et al. showed that overexpression of the miRNA-23a~27a~24–2 cluster is related to increased apoptosis in HEK293T cells due to targeting of the Fas Associated protein with Death Domain (FADD) [47]. Furthermore, miRNA-23a has been found to mediate cardiac hypertrophy, indicating that over-expression of miRNA-23a can initiate hypertrophic signaling [48]. In our present study, we observed that miRNA-23a and miRNA-92a induced cardiac apoptosis and that inhibition of the ischemia-induced upregulation of miRNA-23a and miRNA-92a decreased cardiac apoptosis. Further studies on the targets of miRNA-23a and miRNA-92a are required to determine the underlying mechanisms involved apoptotic cardiomyocytes in MI.

Since miRNAs have been implicated as crucial regulators of gene expression associated with MI and remodeling [49], miRNA expression might alter during the various biological processes in MI. Indeed, several miRNAs are dysregulated at different time points at 3 days and 14 days after MI in both the border zone and remote zone [50]. Similarly, Shi et al. reported that miRNA expression is altered in the infarcted myocardium of rats at 2 days, 7 days, and 14 days after MI, suggesting that altered expression of miRNAs is associated with progression of MI [51]. Port et al. demonstrated that expression of miRNAs changes temporally in response to MI, showing different expression patterns of miRNAs in 2 days, 2 weeks, and 2 months post-MI in a mouse model [52]. In this regard, they suggested that the temporally altered expression of miRNAs correlated with multiple stages post-MI, such as early stage apoptosis or later stage growth promotion. Interestingly, our data indicated that expression of both miRNA-23a and miRNA-92a in the saline-treated group is upregulated at 3 days, but does not increase consistently 4 weeks after saline treatment post-MI (Fig 2). Additionally, we identified differences in the expression of both miRNAs at 3 days between the saline- and cell-treated group (Fig 2). This indicates that the MI-induced transient aberrant expression of miRNAs is possibly rescued by the transplatned BM-MSCs.

**Fig 7:**
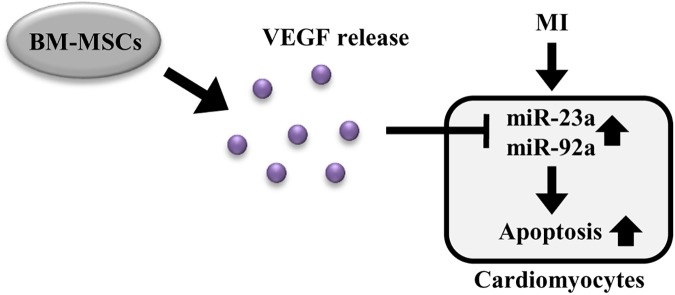
Proposed mechanisms of the paracrine regulation of cardiac miRNAs by transplanted BM-MSCs. Transplanted BM-MSCs release VEGF, which inhibits the MI-induced miRNA-23a and miRNA-92a, thereby blocking cardiomyocytes apoptosis.

Recent studies have demonstrated that transplanted stem cell-derived paracrine factors can modulate the expression of miRNAs in cardiac cells, resulting in improved effects on cardiac diseases. For example, bone marrow progenitor cell-released HGF inhibited miRNA-155-induced fibrosis, thereby exerting an anti-fibrotic effect [53]. In another study, bone marrow mononuclear cell-released insulin-like growth factor (IGF)-1 inhibited the expression of pro-apoptotic miRNA-34a, thereby exerting an anti-apoptotic effect [15]. Similarly, our study showed that MSC-released VEGF inhibited the expression of miRNA-23a and miRNA-92a, which induced cardiac apoptosis, thereby inhibiting ischemic injury-induced cardiac apoptosis (Figs 6 and 7). Additionally, unlike previous studies, we investigated the idealized paracrine factors and miRNAs as candidates through Luminex analysis in BM-MSC-conditioned media and real-time PCR after miRNA microarray in MSC-transplanted heart tissue, respectively.

In this study, we showed that VEGF acts upstream of the miRNA-23a and miRNA-92a inhibition pathway in cardiomyocytes, and plays a key role in the therapeutic effects of MSC transplantation in a rat model of MI (Fig 6). Unfortunately, we were unable to demonstrate a definite mechanism of interaction between paracrine factors and miRNAs. Stem cell-derived paracrine factors are involved in various mechanisms such as alterations in gene expression following transcription factor activation by paracrine factor-mediated intracellular signaling [54]. The expression of miRNAs could also be affected by transcription factors. In fact, miRNAs are known to be encoded from their own promoters inside exonic regions or introns of coding transcripts [42, 55]. In addition, it has been reported that expression of miRNAs could be regulated on multiple levels, including the transcriptional and post-transcriptional level [42]. Thus, the interaction between paracrine factors and miRNAs is complex and remain unclear. We believe that understanding of this cross-talk is critical and therefore, we are planning further studies to investigate this.

The present study has several limitations. First, we were not able to investigate the detailed mechanisms regarding the role of miRNA-23a and miRNA-92a in cardiomyocytes, indicating downregulation of miRNAs by the inhibitory effects of VEGF. Further studies are required to understand the mechanisms involved in the effects of miRNA-23a and miRNA-92a, specifically regarding the occurrence of apoptosis in cardiomyocytes. Second, We could not establish inhibition of cardiac miRNA-23a and miRNA-92a expression in vivo by MSC-derived VEGF. To clarify this, further studies involving the treatment of an MSC-transplanted rat model with anti-VEGF neutralizing antibodies should be performed to confirm the restoration of miRNA-23a and miRNA-92a expression and subsequent apoptosis of cardiomyocytes after VEGF neutralization. Third, echocardiography was not performed to evaluate the cardiac function in a rat model of MI after or before treatment of MSCs. Finally, the number of animals included in the present study was small; therefore, future studies should involve larger number of animals.

## Conclusion

In conclusion, MSC therapy reduced fibrosis and apoptosis of the myocardium in a rat model of MI. These therapeutic effects of MSC therapy could be mediated by MSC-released paracrine factors including VEGF, which inhibited MI-induced upregulation of miRNA-23a and miRNA-92a. MSC-released VEGF inhibited miRNA-23a and miRNA-92a expression, thereby improving cardiac apoptosis in the rat model of MI. This is the first study to show that the therapeutic effects of MSCs in a rat model of MI are mediated by a reduction in the expression of pro-apoptotic miRNAs such as miRNA-23a and miRNA-92a by MSC-released VEGF. Thus, our findings suggest that paracrine factors and miRNAs could be used to develop novel therapeutic drugs for treating patients with MI.

## Supporting information

S1 Fig Four weeks after BM-MSC transplantation, fluorescence microscopy detected BM-MSCs within the infarct area of the myocardium. Frozen sections of heart tissues were stained with DAPI nuclear stain (blue). Representative images of non-transplanted myocardium (A) and BM-MSCs (CM-DiI-positive cells, red) transplanted myocardium (B). a: Magnification, ×200; Scale bar, 100 μm. b: Magnification, ×400; Scale bar, 50 μm.
(TIF)

S2 Fig TUNEL-positive cells in the myocardium after BM-MSC therapy. (A) Representative images showing apoptotic cells for each group in the peri-infarct region. Scale bar, 50 μm. (B) Higher magnification views of the square labelled in (A). Dark green arrow indicates the TUNEL-positive nuclei.
(TIF)

S3 Fig Microarray analysis of miRNA expression in the infarcted myocardium. The miRNA clustering tree of the BM-MSC-treated group and saline-treated group. The color scale illustrates the relative expression level of miRNAs and, specifically, red represents an expression level higher than the saline-treated group, whereas blue represents an expression level lower than the saline-treated group. This heat map diagram shows the expression of the 39 different miRNAs; 5 of these miRNAs were significantly higher than the saline-treated group and 34 miRNAs were significantly lower than the saline-treated group.
(TIF)

S4 Fig Validation of expression of candidate miRNAs selected by microarray data. Candidate miRNAs expression was measured by real-time PCR using Taqman probes in order to confirm the validation of microarray data. MiRNA-21 (A), miRNA-199a (B), miRNA-130b (C), miRNA-138-1 (D), miRNA-9 (E), miRNA-27a (F), miRNA-125a (G), and miRNA-320 (H) expression was not validated at 3 days after treatment with BM-MSC. All data are expressed as mean ± SD (n = 5 per group). *P < 0.05 vs. sham control group.
(TIF)

S5 Fig MCP-1 released from BM-MSCs has no effect on miRNA-23a and miRNA-92a expression and apoptosis in vitro. MiRNA-23a (A) and miRNA-92a (B) expression was not regulated by depending on presence or absence of MCP-1 in BM-MSCs hypoxic-conditioned media. Expression of miRNA was determined by real-time PCR using TaqMan probes. (C) The presence or absence of MCP-1 in BM-MSCs hypoxic-conditioned media was not related to apoptosis of cardiomyocytes. MSCs media indicates hypoxia-exposed BM-MSC-conditioned media. Quantitative analysis of apoptotic cells was measured by annexin V staining. All data are expressed as mean ± SD (*n* = 5 per group). *P < 0.05 vs. normoxia without netralizing antibodies against MCP-1 (MCP-1 ab). ^&^P < 0.05 vs. normoxia with MCP-1 ab.
(TIF)

S1 Table Information for TaqMan® MicroRNA assay. (DOCX)

S2 Table Sequences of miRNA inhibitors. (DOCX)

S3 Table Luminex screening assay. (DOCX)
