# Supplementary figures and images for: Bone marrow mesenchymal stem cell-derived vascular endothelial growth factor attenuates cardiac apoptosis via regulation of cardiac miRNA-23a and miRNA-92a in a rat model of myocardial infarction
Source: PLoS One. 2017 Jun 29;12(6):e0179972. doi: 10.1371/journal.pone.0179972 (PMC5491110; doi:10.1371/journal.pone.0179972)

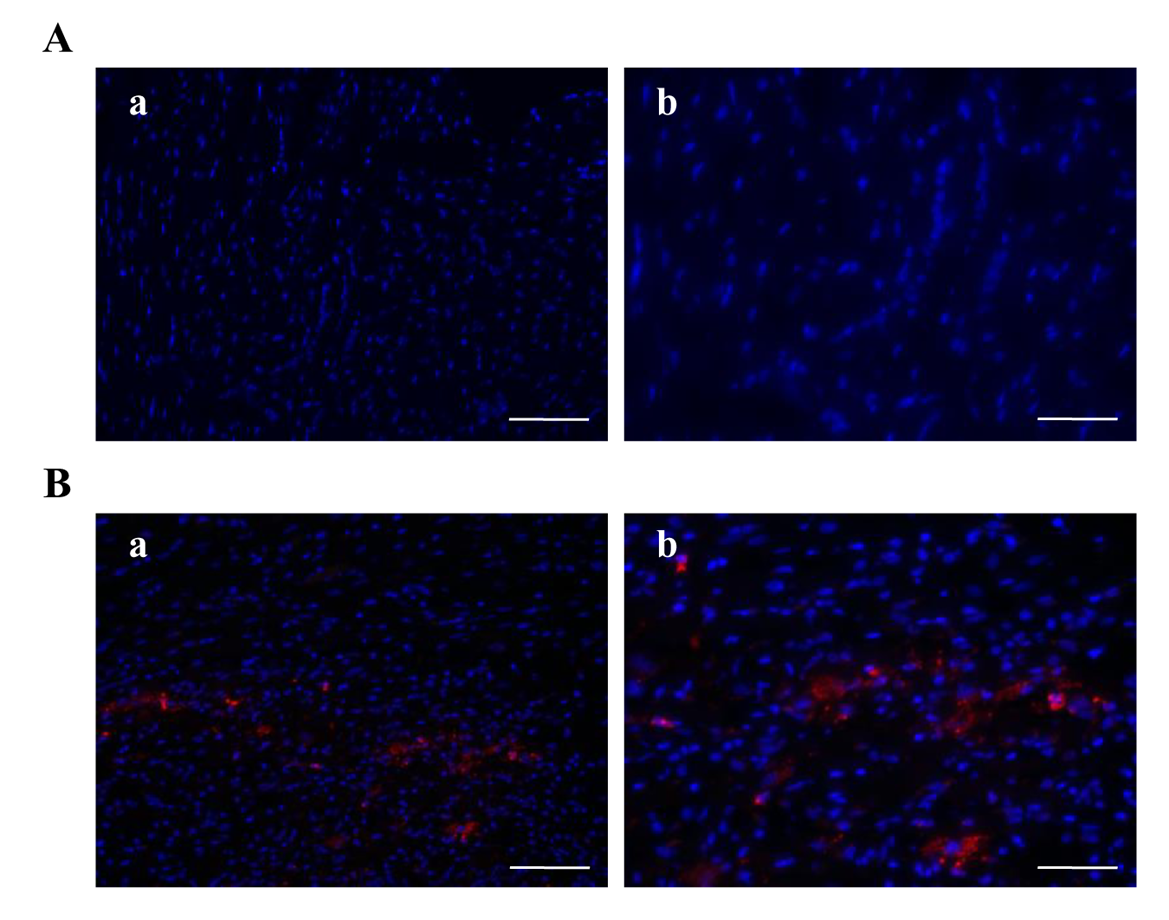

Supplement: S1 Fig — Frozen sections of heart tissues were stained with DAPI nuclear stain (blue). Representative images of non-transplanted myocardium (A) and BM-MSCs (CM-DiI-positive cells, red) transplanted myocardium (B). a: Magnification, ×200; Scale bar, 100 μm. b: Magnification, ×400; Scale bar, 50 μm. (TIF) [file pone.0179972.s001.TIF]

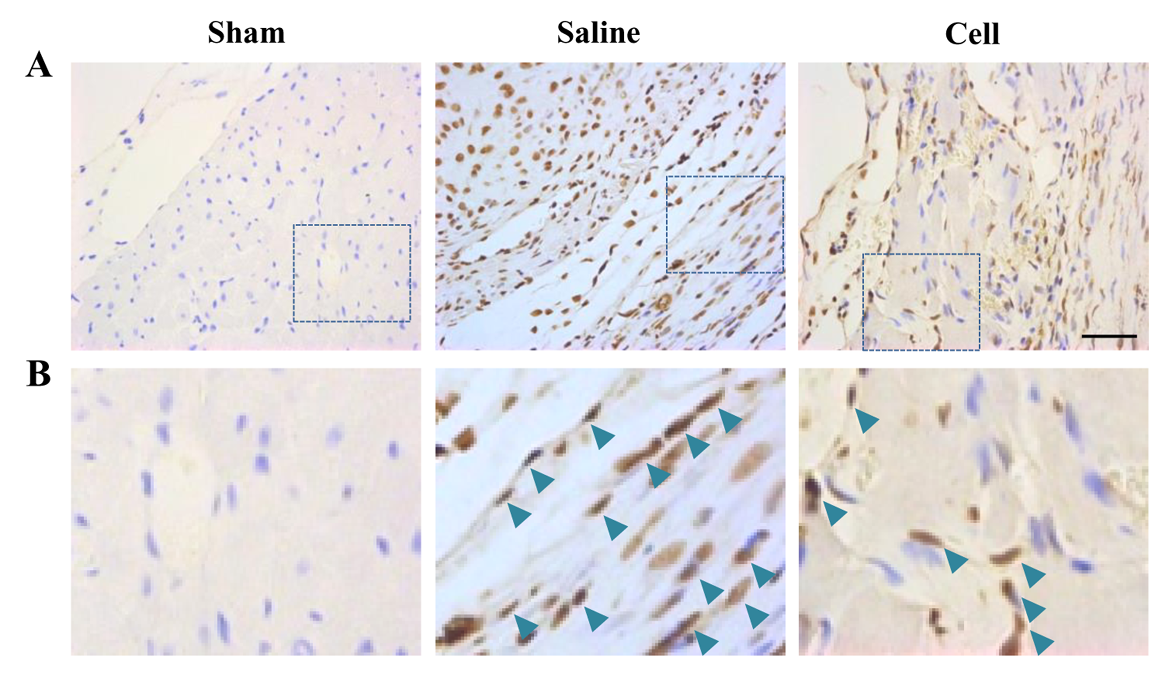

Supplement: S2 Fig — (A) Representative images showing apoptotic cells for each group in the peri-infarct region. Scale bar, 50 μm. (B) Higher magnification views of the square labelled in (A). Dark green arrow indicates the TUNEL-positive nuclei. (TIF) [file pone.0179972.s002.TIF]

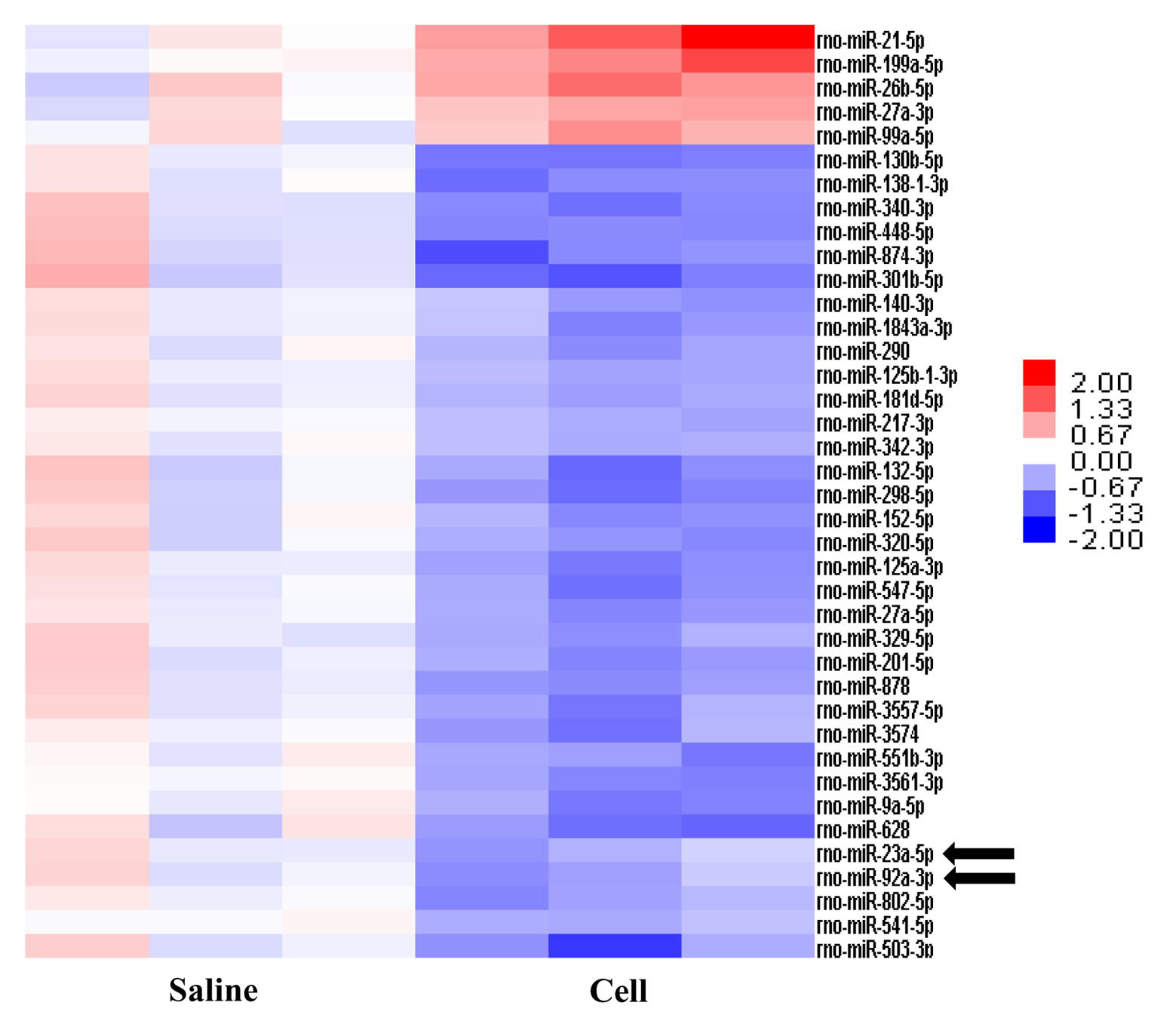

Supplement: S3 Fig — The miRNA clustering tree of the BM-MSC-treated group and saline-treated group. The color scale illustrates the relative expression level of miRNAs and, specifically, red represents an expression level higher than the saline-treated group, whereas blue represents an expression level lower than the saline-treated group. This heat map diagram shows the expression of the 39 different miRNAs; 5 of these miRNAs were significantly higher than the saline-treated group and 34 miRNAs were significantly lower than the saline-treated group. (TIF) [file pone.0179972.s003.TIF]

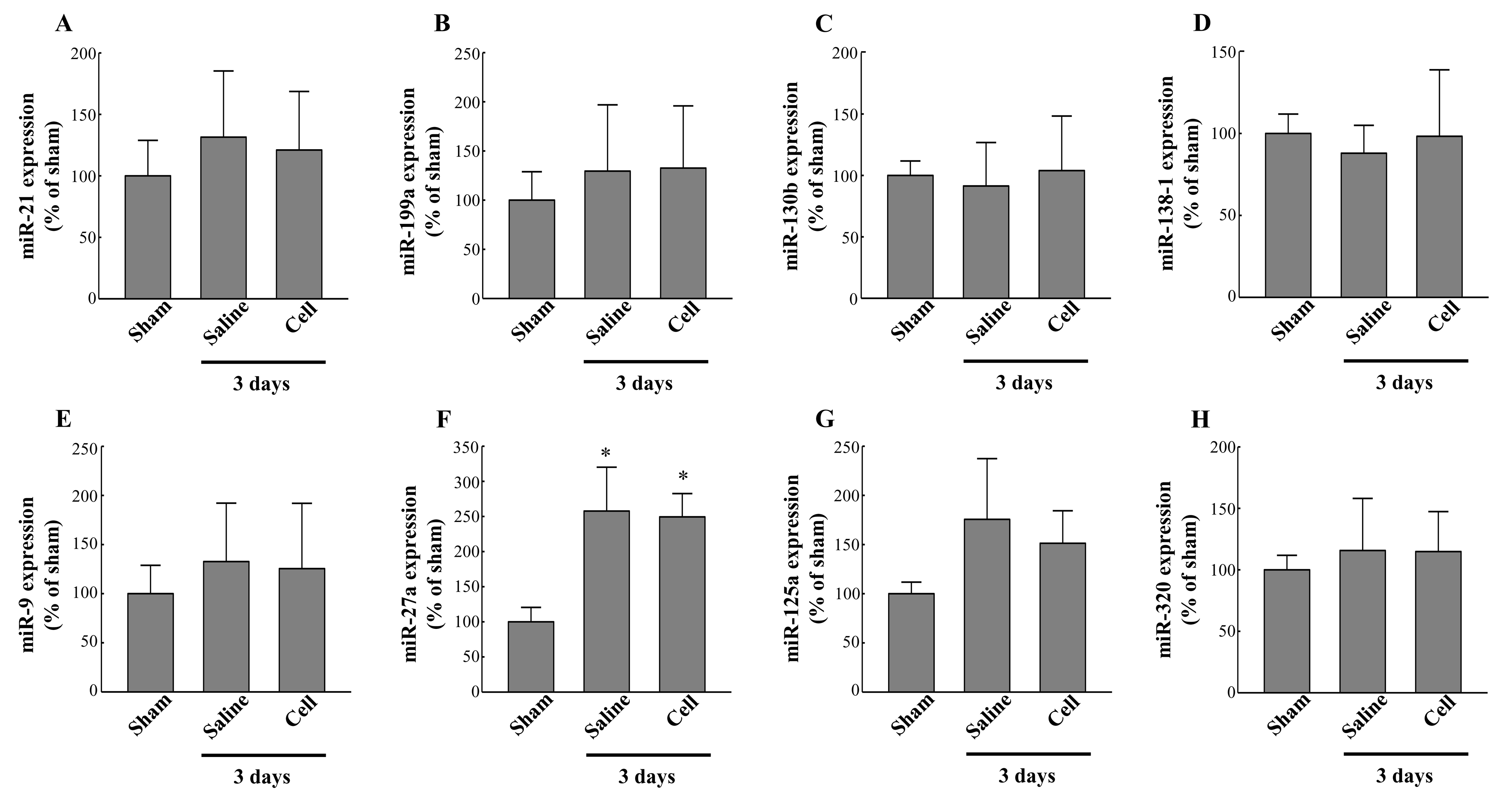

Supplement: S4 Fig — Candidate miRNAs expression was measured by real-time PCR using Taqman probes in order to confirm the validation of microarray data. MiRNA-21 (A), miRNA-199a (B), miRNA-130b (C), miRNA-138-1 (D), miRNA-9 (E), miRNA-27a (F), miRNA-125a (G), and miRNA-320 (H) expression was not validated at 3 days after treatment with BM-MSC. All data are expressed as mean ± SD (n = 5 per group). *P < 0.05 vs. sham control group. (TIF) [file pone.0179972.s004.TIF]

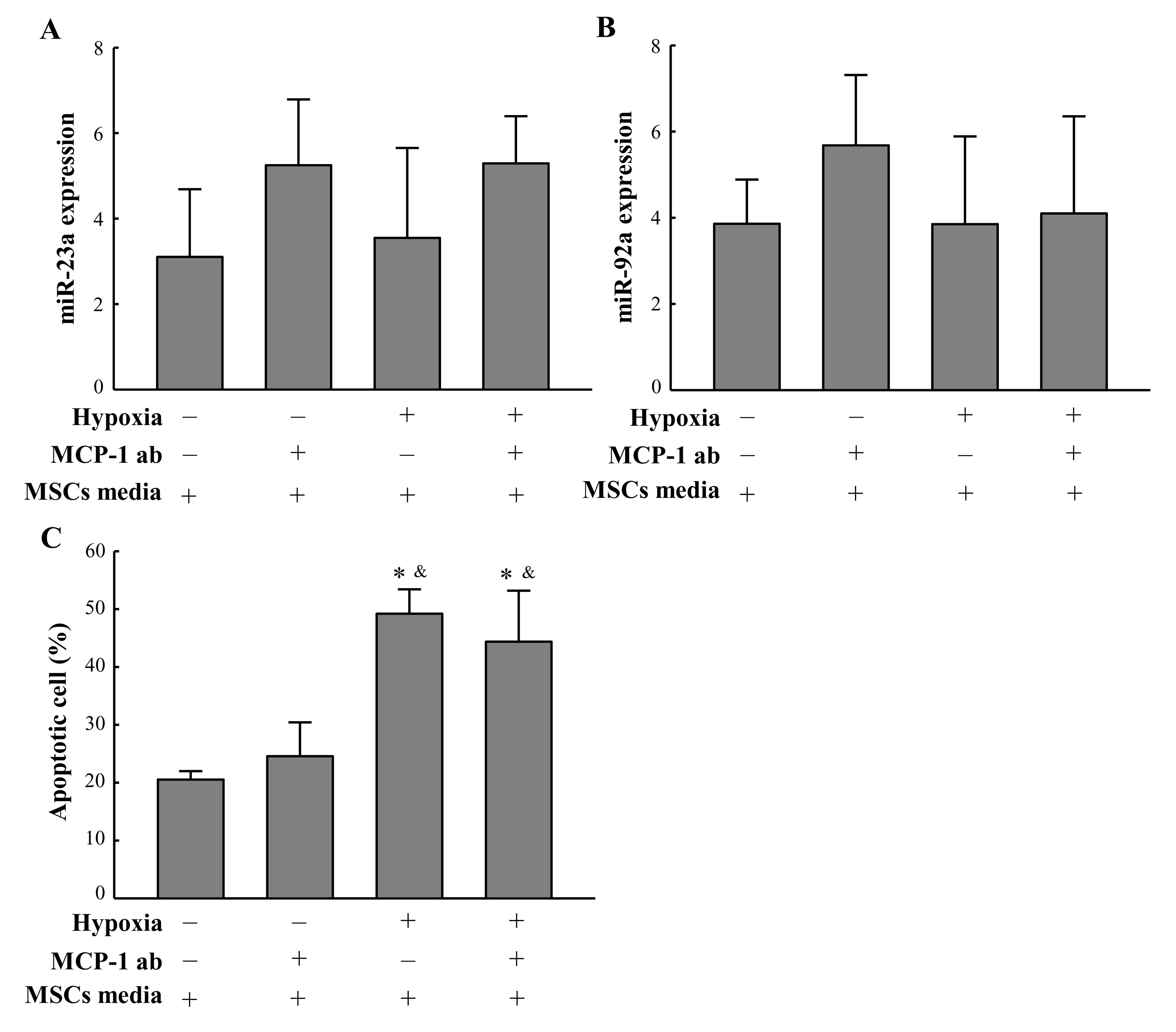

Supplement: S5 Fig — MiRNA-23a (A) and miRNA-92a (B) expression was not regulated by depending on presence or absence of MCP-1 in BM-MSCs hypoxic-conditioned media. Expression of miRNA was determined by real-time PCR using TaqMan probes. (C) The presence or absence of MCP-1 in BM-MSCs hypoxic-conditioned media was not related to apoptosis of cardiomyocytes. MSCs media indicates hypoxia-exposed BM-MSC-conditioned media. Quantitative analysis of apoptotic cells was measured by annexin V staining. All data are expressed as mean ± SD (n = 5 per group). *P < 0.05 vs. normoxia without netralizing antibodies against MCP-1 (MCP-1 ab). &P < 0.05 vs. normoxia with MCP-1 ab. (TIF) [file pone.0179972.s005.TIF]
