# Supplementary material for: Bone marrow mesenchymal stem cell-derived vascular endothelial growth factor attenuates cardiac apoptosis via regulation of cardiac miRNA-23a and miRNA-92a in a rat model of myocardial infarction
Source: PLoS One. 2017 Jun 29;12(6):e0179972. doi: 10.1371/journal.pone.0179972 (PMC5491110; doi:10.1371/journal.pone.0179972)
Supplement: S1 Table — (DOCX) [file pone.0179972.s006.docx]

**S1 Table. Information for TaqMan® MicroRNA assay.**

| **Assay name** | **Assay ID** | **Target sequence (5' to 3')** |
| --- | --- | --- |
| miRNA-23a | 000399 | AUCACAUUGCCAGGGAUUUCC |
| miRNA-92a | 000431 | UAUUGCACUUGUCCCGGCCUGU |
| RNU6B (U6) | 001093 | CGCAAGGAUGACACGCAAAUUC-  GUGAAGCGUUCCAUAUUUUU |
