# Supplementary material for: Bone marrow mesenchymal stem cell-derived vascular endothelial growth factor attenuates cardiac apoptosis via regulation of cardiac miRNA-23a and miRNA-92a in a rat model of myocardial infarction
Source: PLoS One. 2017 Jun 29;12(6):e0179972. doi: 10.1371/journal.pone.0179972 (PMC5491110; doi:10.1371/journal.pone.0179972)
Supplement: S2 Table — (DOCX) [file pone.0179972.s007.docx]

**S2 Table. Sequences of miRNA inhibitors.**

| **Name** | **Inhibitor sequence (5' to 3')** |
| --- | --- |
| miRNA-23a inhibitor | GGAAAUCCCUGGCAAUGUGAU |
| miRNA-92a inhibitor | ACAGGCCGGGACAAGUGCAAUA |
| Negative Control (NC) | CAGUACUUUUGUGUAGUACAA |
