# Supplementary material for: Bone marrow mesenchymal stem cell-derived vascular endothelial growth factor attenuates cardiac apoptosis via regulation of cardiac miRNA-23a and miRNA-92a in a rat model of myocardial infarction
Source: PLoS One. 2017 Jun 29;12(6):e0179972. doi: 10.1371/journal.pone.0179972 (PMC5491110; doi:10.1371/journal.pone.0179972)
Supplement: S3 Table — (DOCX) [file pone.0179972.s008.docx]

**S3 Table. Luminex screening assay.**

| **Paracrine factor**  **(pg/ml)** | **Normoxia**  **(48 hours)** | **Hypoxia**  **(48 hours)** |
| --- | --- | --- |
| Adiponectin | 0.00 ± 0.00 | 0.00 ± 0.00 |
| ANG | 43.72 ± 10.42 | 95.01 ± 4.71* |
| BDNF | 0.00 ± 0.00 | 0.00 ± 0.00 |
| EGF | 0.55 ± 0.00 | 0.57 ± 0.05 |
| FGF-21 | 773.69 ± 28.02 | 865.78 ± 21.2 |
| G-CSF | 0.00 ± 0.00 | 0.00 ± 0.00 |
| GDNF | 0.04 ± 0.06 | 0.05 ± 0.11 |
| GM-CSF | 0.00 ± 0.00 | 0.00 ± 0.00 |
| HGF | 0.00 ± 0.00 | 0.00 ± 0.00 |
| IL-6 | 4.51 ± 1.58 | 22.49 ± 5.03* |
| Leptin | 0.00 ± 0.00 | 0.00 ± 0.00 |
| MCP-1 | 93.32 ± 58.27 | 329.22 ± 36.62* |
| VEGF | 199.28 ± 83.1 | 431.89 ± 30.25* |

ANG, angiogenin; BDNF, brain-derived neurotrophic factor; EGF, epidermal growth factor; FGF-21, fibroblast growth factor-21; G-CSF, granulocyte-colony stimulating factor; GDNF, glial cell-derived neurotrophic factor; GM-CSF, granulocyte macrophage-colony stimulating factor; HGF, hepatocyte growth factor; IL-6, interleukin-6; MCP-1, monocyte chemoattractant protein-1; VEGF, vascular endothelial growth factor. *P < 0.05 vs. Normoxia group
